# Population norms for the EQ-5D-3L and EQ-5D-5L in Peninsular Malaysia

**DOI:** 10.1007/s11136-026-04231-x

**Published:** 2026-04-01

**Authors:** Annushiah Vasan Thakumar, Xun Li, Asrul Akmal Shafie, Ling Jie Cheng

**Affiliations:** 1 School of Pharmacy, Faculty of Health and Medical Sciences, Taylor’s University, Subang Jaya, Malaysia; 2 School of Engineering, Computing, and Mathematics, Oxford Brookes University, Oxford, United Kingdom; 3 Discipline of Social and Administrative Pharmacy, School of Pharmaceutical Sciences, Universiti Sains Malaysia, George Town, Malaysia; 4 National Perinatal Epidemiology Unit, Nuffield Department of Women’s and Reproductive Health, University of Oxford, Oxford, United Kingdom; 5 Alice Lee Centre for Nursing Studies, Yong Loo Lin School of Medicine, National University of Singapore, Singapore, Singapore

**Keywords:** EQ-5D-3L, EQ-5D-5L, Population norms, Health-related quality of life, Malaysia, EQ VAS

## Abstract

**Purpose:**

Population norms for preference-based health-related quality of life (HRQoL) instruments provide essential reference data for burden-of-disease assessments, economic evaluations, and quality-adjusted life year (QALY) calculations. Although Malaysian EQ-5D value sets are available, population norms have not been established. This study aimed to establish the first population norms for the EQ-5D-3L and EQ-5D-5L in Peninsular Malaysia, compare their distributional characteristics, and identify factors associated with HRQoL.

**Methods:**

We conducted a cross-sectional study among 1,137 adults aged 18 years or older across eight locations in four regional clusters of Peninsular Malaysia. Quota-based sampling was stratified by urbanicity, sex, age, and ethnicity. Participants completed the EQ-5D-5L, EQ-5D-3L, and EQ visual analogue scale (EQ VAS). We calculated index scores using Malaysian value sets and constructed survey weights using iterative proportional fitting to the 2020 Malaysian census. We estimated population norms overall and by sociodemographic strata. Multivariable regression analyses identified factors independently associated with HRQoL.

**Results:**

The weighted mean EQ-5D-5L index score was 0.919 (SD 0.105), the EQ-5D-3L index score was 0.945 (SD 0.090), and the EQ VAS score was 85.4 (SD 12.5). The ceiling effect was substantially lower for the EQ-5D-5L than for the EQ-5D-3L (44.4% vs. 68.5%), with 96 and 29 unique health profiles observed, respectively. Pain/discomfort and anxiety/depression were the most frequently reported problems. Older age, unemployment, rural residence, and doctor-diagnosed disease were associated with lower HRQoL.

**Conclusion:**

This study provides the first population norms for the EQ-5D-3L and EQ-5D-5L in Peninsular Malaysia. These norms serve as essential reference values for clinical research, economic evaluations, and health policy decision-making.

**Supplementary Information:**

The online version contains supplementary material available at 10.1007/s11136-026-04231-x.

## Abstract (plain language summary)

This study assessed the overall health of adults in Peninsular Malaysia using two standardised questionnaires, the EQ-5D-3L and EQ-5D-5L. These questionnaires ask people to rate their health across five areas: mobility, self-care, usual activities, pain or discomfort, and anxiety or depression. We interviewed 1,137 adults aged 18 years and older from different parts of the country. We combined their responses into single health scores and adjusted the results to represent the wider Malaysian adult population using national census data. Overall, Malaysian adults reported good health, with an average EQ-5D-5L score of 0.919 out of 1.0. Pain or discomfort was the most commonly reported problem (36%), followed by anxiety or depression (28%). Older adults, people living in rural areas, and those with a doctor-diagnosed disease tended to report poorer health. We also found that the newer five-level version of the questionnaire (EQ-5D-5L) was better at detecting differences in health than the older three-level version (EQ-5D-3L), capturing more than three times as many unique health profiles. These reference values, known as population norms, allow researchers and policymakers to compare the health of specific patient groups with that of the general Malaysian population, supporting clinical research and healthcare decision-making.

**Supplementary Information:**

The online version contains supplementary material available at 10.1007/s11136-026-04231-x.

## Introduction

Health-related quality of life (HRQoL) is an increasingly important outcome in healthcare decision-making, clinical research, and population health monitoring [1]. In health technology assessment (HTA), preference-based HRQoL measures are particularly important because they generate utility values for calculating quality-adjusted life years (QALYs), which underpin cost-utility analyses [2]. Among available generic preference-based instruments, the EQ-5D, developed by the EuroQol Group, is one of the most widely used measures of HRQoL worldwide [3–6].

The EQ-5D includes a descriptive system that classifies health across five dimensions: mobility (MO), self-care (SC), usual activities (UA), pain/discomfort (PD), and anxiety/depression (AD). The original three-level version (EQ-5D-3L) defines three response levels (no problems, some problems, and extreme problems), resulting in 243 possible health states [7]. To address the ceiling effect and limited sensitivity of the EQ-5D-3L, particularly in milder health states, the EuroQol Group introduced the five-level version (EQ-5D-5L) in 2011. This version expands response options to five levels and defines 3,125 health states [8]. Both instruments include the EQ visual analogue scale (EQ VAS), on which respondents rate their overall health from 0 (worst imaginable) to 100 (best imaginable) [9]. Comparative studies across multiple countries have shown that the EQ-5D-5L demonstrates improved measurement properties, including reduced ceiling effects, greater discriminatory power, and better convergent validity [10, 11].

EQ-5D health profiles can be converted into a single summary index score using country-specific value sets that reflect general population preferences for different health state [12]. These value sets enable the estimation of population norms, which are reference values derived from representative population samples. Population norms allow comparison between patient groups and the general population and support disease burden estimation, economic evaluation, and health policy decision-making [13].

Population norms for the EQ-5D have been established in many countries. Janssen et al. [13] published the first international compendium of EQ-5D norms, followed by a large multi-country study of EQ-5D-3L norms across 20 countries (*N* = 163,838) [14]. EQ-5D-5L norms have since been reported for an increasing number of settings, including the United States [15], Poland [16], South Australia [17], and Trinidad and Tobago [18]. A recent study across 23 countries reported a mean utility score of 0.897 and a mean EQ VAS of 79.0, with systematic variation by region, age, and sex [19]. In Asia, EQ-5D-5L population norms have been published for Japan [20], urban China [21], Indonesia [22], Vietnam [23], Singapore [24], Hong Kong [25], and Thailand [26]. Notably, the Thai study reported norms for both the EQ-5D-3L and EQ-5D-5L, enabling direct comparison between instruments [26]. Across studies, self-reported health consistently declines with age, is generally lower among females, and varies by sociodemographic characteristics [14, 20, 27].

Malaysia has developed a relatively mature HTA infrastructure over the past two decades. The Malaysian Pharmacoeconomic Guidelines, first published in 2012 and updated in 2019, recommend QALYs as the primary outcome for cost-utility analyses and emphasise the use of utility values derived from the local population [28]. The EQ-5D has been validated in Malaysia. Shafie et al. [29] demonstrated the construct validity of the EQ-5D-3L in a northern Malaysian sample. More recently, evidence from a national sample showed that the EQ-5D-5L had superior psychometric performance compared with the EQ-5D-3L, including reduced ceiling effects and improved informativity and convergent validity [30]. A Malaysian EQ-5D-5L value set was subsequently developed using a nationally representative sample and the EuroQol Valuation Technology protocol, applying a hybrid model that combined composite time trade-off and discrete choice experiment data [31]. In addition, a Malaysian EQ-5D-3L value set, based on time trade-off and visual analogue scale methods, is available [32]. Together, these developments provide a robust methodological foundation for estimating population norms.

Despite the availability of validated instruments and national value sets, population norms for the EQ-5D have not yet been established in Malaysia. This gap limits the interpretation of utility scores and reduces the usefulness of local utility data for economic evaluation and international comparison. Establishing population norms for both the EQ-5D-3L and EQ-5D-5L within the same population is particularly valuable during the ongoing transition between instruments. This approach allows direct comparison of distributional properties and ceiling effects, facilitates mapping between versions, and supports evidence synthesis across studies using different EQ-5D instruments [30]. Although the EQ-5D-5L offers improved measurement properties [10, 11, 30], a substantial body of existing evidence and some HTA reference case requirements remain based on the EQ-5D-3L [33]. Population norms for both versions therefore improve comparability across studies and support evidence synthesis [33].

This study aimed to establish the first population norms for the EQ-5D-3L and EQ-5D-5L in Peninsular Malaysia. The specific objectives were:

1. To estimate population norms for the EQ-5D-5L index, EQ-5D-3L index, and EQ VAS scores, weighted to the 2020 Malaysian census population structure and stratified by key sociodemographic characteristics;
2. To compare the distributional characteristics of the EQ-5D-5L and EQ-5D-3L when administered to the same population, including ceiling effects, number of unique health profiles, and dimension-level problem reporting;
3. To identify sociodemographic and health-related factors independently associated with health-related quality of life;
4. To assess the robustness of the estimated norms using alternative census weighting approaches.

## Methods

### Study design and setting

This was a cross-sectional, population-based study conducted across Peninsular Malaysia. The study was undertaken in parallel with the Malaysian EQ-5D-5L valuation study [31], which used the same sampling framework and data collection procedures. Data were collected between August and October 2016.

### Sampling strategy

The study targeted 1,200 Malaysian adults to account for potential attrition. Peninsular Malaysia’s 11 states were grouped into four regional clusters (Northern, Central, Southern, and East Coast), and two locations (one urban, one rural) were selected randomly from each cluster using simple random sampling, yielding eight data collection sites. Within each site, quota-based sampling was employed to match the Malaysian census population structure on urbanicity, gender, age group, and ethnicity. Interviewers approached potential participants in high-traffic public areas and in residential settings to recruit a diverse and representative sample.

## Participants

Eligible participants were adults aged 18 years and older who were able to understand and communicate in English or Malay. Individuals with cognitive impairment or communication difficulties that precluded self-report were excluded. All participants provided written informed consent prior to data collection.

## Data collection procedures

Data were collected through face-to-face interviews conducted by trained interviewers. Each respondent first completed the EQ-5D-5L descriptive system, followed by a single EQ VAS rating, and then the EQ-5D-3L descriptive system. The EQ VAS was administered once per respondent as part of the EQ-5D-5L assessment and was not repeated for the EQ-5D-3L. Sociodemographic information was collected on age, sex, ethnicity (Malay, Chinese, Indian, other), state of residence, area of residence (urban or rural), highest education level, employment status, marital status, household income, household size, and number of income earners in the household. Respondents also reported whether they had been diagnosed with any disease by a doctor and rated their general health.

## EQ-5D scoring

EQ-5D-5L health profiles were converted into index scores using the Malaysian EQ-5D-5L value set [31], which was derived using a constrained eight-parameter hybrid model combining C-TTO and DCE data. EQ-5D-3L health profiles were scored using the Malaysian EQ-5D-3L VAS-based value set [32], which employs a constant term (0.067), dimension-specific disutility decrements for levels 2 and 3, and an N3 penalty (0.116) applied when any dimension is at level 3. Index scores for both instruments were computed from the individual dimension responses for all respondents with complete data. The EQ VAS was recorded directly as reported by respondents on a scale from 0 to 100 [9].

### Statistical analysis

To ensure population representativeness, survey weights were constructed using iterative proportional fitting (raking) [34] on four marginal distributions: sex, age group (18–24, 25–34, 35–44, 45–54, 55–64, and ≥ 65 years), ethnicity (Malay, Chinese, Indian, and other), and area of residence (urban and rural). Age proportions from the census were recalculated for the adult population aged ≥ 18 years. The primary analysis used the 2020 Malaysian census [35] as the target population structure. A sensitivity analysis was conducted using the 2010 Malaysian census [36] to assess the robustness of the norms to changes in population structure. Weight diagnostics showed acceptable properties (coefficient of variation: 0.35 for the 2020 weights, 0.23 for the 2010 weights; range: 0.38–2.21).

Weighted descriptive statistics were calculated for the EQ-5D-5L index, EQ-5D-3L index, and EQ VAS, including means, standard deviations (SDs), and medians. Population norms were estimated for the total sample and stratified by age group, sex, ethnicity, and area of residence. The proportion of respondents reporting any problem (levels 2–5 for EQ-5D-5L; levels 2–3 for EQ-5D-3L) was calculated for each dimension. The ceiling effect was defined as the proportion reporting full health (profile 11111). The number of unique health profiles was compared between instruments to assess discriminatory capacity.

Multivariable regression analyses were conducted to identify sociodemographic factors independently associated with EQ-5D-5L index, EQ-5D-3L index, and EQ VAS scores. For the EQ-5D index scores, both ordinary least squares (OLS) regression with heteroscedasticity-consistent (HC1) standard errors and upper-censored Tobit regression were employed. The Tobit model was used to account for the substantial ceiling effect (i.e., a large proportion of observations at the upper bound of 1.0), as OLS estimates are biased towards zero when the dependent variable is censored [37, 38]. Different regression approaches were selected to match the distributional characteristics of each outcome. The EQ-5D index scores exhibit substantial ceiling effects (i.e. a large proportion of observations at the upper bound of 1.0), motivating the use of Tobit models alongside OLS. In contrast, the EQ VAS is continuous and right-skewed without a boundary mass, making a GLM sensitivity analysis more appropriate than a Tobit model. For the EQ VAS, OLS regression with robust standard errors was used as the primary model, complemented by a generalised linear model (GLM) with a Gamma family and log-link function as a sensitivity analysis, given the bounded and right-skewed nature of VAS scores [39]. The choice of Gamma family and log-link function for the GLM was guided by theoretical considerations and empirical specification tests, including the modified Park test for the distributional family and the Pregibon link test for the link function. Independent variables included age group (reference: 18–24), sex (reference: male), ethnicity (reference: Malay), area of residence (reference: urban), highest education level (reference: tertiary), employment status (reference: employed), marital status (reference: married/cohabiting), and presence of doctor-diagnosed disease (reference: no disease). All analyses were conducted using Stata version 19.0 (StataCorp LLC, College Station, TX, USA) [40].

## Ethical considerations

The study received ethical approval from the Malaysia Medical Research & Ethics Committee (ID: NMRR-13-1377-18574) and was conducted in accordance with the Declaration of Helsinki. Written informed consent was obtained from all participants.

## Results

### Sample characteristics

Of the 1,172 individuals who provided informed consent, 1,137 (97.0%) successfully completed interviews and were included in the analysis. The mean age was 39.0 years (range: 18–88). The sample comprised 584 (51.4%) males and 553 (48.6%) females. The majority were Malay (*n* = 772, 67.9%), followed by Chinese (*n* = 288, 25.3%), Indian (*n* = 67, 5.9%), and other ethnicities (*n* = 10, 0.9%). Urban residents comprised 70.3% of the sample (*n* = 799). The largest age group was 18–24 years (*n* = 304, 26.7%), reflecting the relatively young demographic structure of the Malaysian population. Over half (55.8%) were employed or self-employed, with students comprising 20.2%. The majority rated their general health as ‘good’ (57.7%) or ‘very good’ (18.9%), while 21.8% reported ‘fair’ and 0.6% ‘bad’ health. Approximately 28.1% reported at least one doctor-diagnosed disease. Twenty-eight respondents who answered “don’t know” to the disease question were classified as having no diagnosed disease. The sample distribution was broadly comparable to the Malaysian census (Table 1).

**Table 1:** Sociodemographic characteristics of respondents (*N* = 1,137)

Characteristic	*n*	Sample (%)	Census 2020 (%)	Census 2010 (%)
*Sex*				
Male	584	51.4	55.0	51.5
Female	553	48.6	45.0	48.5
*Age group, years*				
18–24	304	26.7	16.8	21.2
25–34	255	22.4	23.7	26.1
35–44	166	14.6	21.9	19.7
45–54	168	14.8	15.3	15.7
55–64	152	13.4	11.4	9.9
≥65	92	8.1	10.9	7.5
*Ethnicity*				
Malay	772	67.9	69.4	67.4
Chinese	288	25.3	23.2	24.5
Indian	67	5.9	6.7	7.3
Other	10	0.9	0.7	0.7
*Area of residence*				
Urban	799	70.3	75.1	71.0
Rural	338	29.7	24.9	29.0
*Education*				
Primary or below	68	6.0	—	—
Secondary	407	35.8	—	—
Tertiary	662	58.2	—	—
*Employment status*				
Employed	647	56.9	—	—
Student	230	20.2	—	—
Retired	117	10.3	—	—
Unemployed/other	134	11.8	—	—
*Marital status*				
Married/cohabiting	589	51.8	—	—
Never married	455	40.0	—	—
Widowed/divorced/separated	82	7.2	—	—
*Doctor-diagnosed disease*				
No	807	71.0	—	—
Yes	319	28.1	—	—
*Self-rated health*				
Bad	7	0.6	—	—
Fair	248	21.8	—	—
Good	656	57.7	—	—
Very good	215	18.9	—	—
*Household income (monthly)*				
< RM3,000	479	42.1	—	—
RM3,000–5,999	375	33.0	—	—
RM6,000–9,999	146	12.8	—	—
≥RM10,000	128	11.3	—	—

RM = Malaysian Ringgit; USD = United States Dollar. USD equivalents based on 2016 average exchange rate (1 USD ≈ 4.15 RM). Census 2020 and Census 2010 refer to Malaysian Population and Housing Census weighted proportions for the adult (≥ 18 years) population. — = not available from census data.

### EQ-5D-5L population norms (2020 census-weighted)

**Fig. 1:**
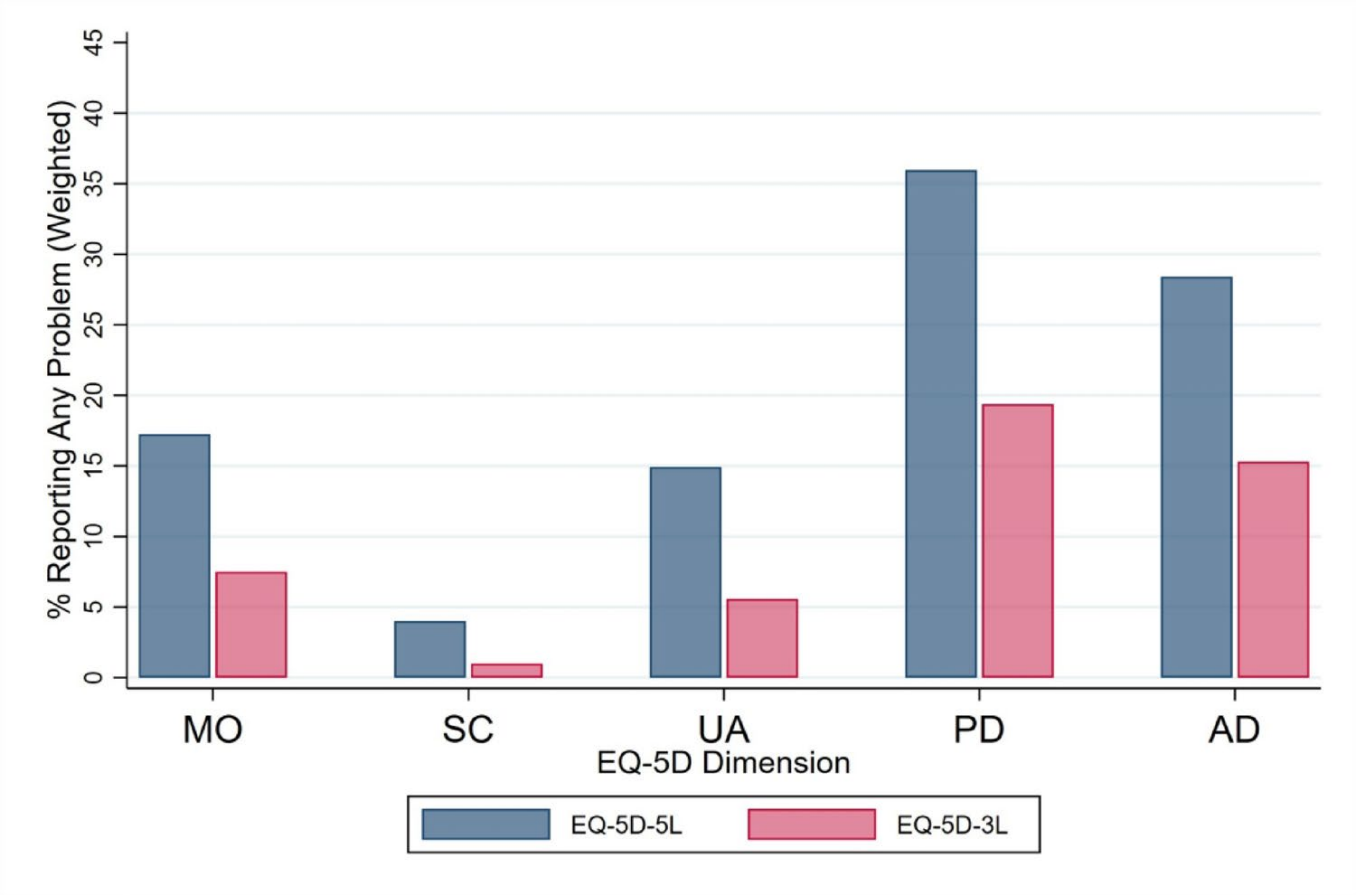
Proportion reporting any problem by EQ-5D dimension: EQ-5D-5L versus EQ-5D-3L (2020 census-weighted). MO = mobility; SC = self-care; UA = usual activities; PD = pain/discomfort; AD = anxiety/depression; EQ-5D-5L = five-level EQ-5D; EQ-5D-3L = three-level EQ-5D.

The weighted mean EQ-5D-5L index score was 0.919 (SD = 0.105), with a median of 0.928 (*n* = 1,137; Supplementary Fig. S1). A total of 44.4% of respondents (weighted) reported full health (profile 11111), and 96 unique health profiles were observed (Table 2). Pain/discomfort was the dimension with the highest prevalence of any reported problem (36.0%), followed by anxiety/depression (28.4%), mobility (17.3%), usual activities (14.9%), and self-care (4.0%) (Fig. 1). The most frequently reported profiles after 11111 were 11121 (11.3%), 11112 (11.1%), and 11122 (4.8%) (Supplementary Table S2).

**Table 2:** Census-weighted EQ-5D-5L population norms by sociodemographic characteristics (2020 Census)

Characteristic	*n*	Mean (SD)	Median	IQR	Ceiling (%)
Total	1,137	0.919 (0.105)	0.928	0.857–1.000	44.4
*Age group,years*					
18–24	304	0.938 (0.081)	1.000	0.919–1.000	50.4
25–34	255	0.935 (0.092)	0.952	0.919–1.000	48.7
35–44	166	0.914 (0.099)	0.928	0.847–1.000	41.5
45–54	168	0.927 (0.094)	0.952	0.880–1.000	48.8
55–64	152	0.901 (0.115)	0.928	0.838–1.000	41.8
≥65	92	0.868 (0.149)	0.919	0.811–1.000	28.3
*Sex*					
Male	584	0.918 (0.103)	0.928	0.857–1.000	44.6
Female	553	0.919 (0.107)	0.928	0.866–1.000	44.2
*Ethnicity*					
Malay	772	0.916 (0.104)	0.928	0.857–1.000	42.0
Chinese	288	0.928 (0.100)	1.000	0.905–1.000	51.3
Indian	67	0.910 (0.123)	0.919	0.871–1.000	43.9
Other	10	0.937 (0.105)	1.000	0.847–1.000	62.7
*Area*					
Urban	799	0.924 (0.099)	0.928	0.871–1.000	46.1
Rural	338	0.903 (0.118)	0.928	0.847–1.000	39.4
*Education*					
Primary or below	68	0.869 (0.144)	0.893	0.811–1.000	33.9
Secondary	407	0.904 (0.115)	0.928	0.847–1.000	39.6
Tertiary	662	0.934 (0.087)	0.952	0.919–1.000	48.9
*Employment*					
Employed	647	0.928 (0.095)	0.938	0.892–1.000	48.1
Student	230	0.934 (0.082)	0.928	0.919–1.000	47.9
Retired	117	0.877 (0.133)	0.919	0.799–1.000	32.0
Unemployed/other	134	0.889 (0.130)	0.919	0.838–1.000	33.2
*Marital status*					
Married/cohabiting	589	0.917 (0.104)	0.928	0.847–1.000	44.9
Never married	455	0.926 (0.096)	0.928	0.893–1.000	45.7
Widowed/divorced/separated	82	0.899 (0.135)	0.928	0.845–1.000	37.6
*Disease*					
No disease	807	0.937 (0.089)	1.000	0.919–1.000	51.9
Has disease	319	0.876 (0.123)	0.919	0.811–1.000	27.8
*Household income*					
< RM3,000	479	0.904 (0.113)	0.928	0.845–1.000	38.6
RM3,000–5,999	375	0.928 (0.100)	0.936	0.905–1.000	48.7
RM6,000–9,999	146	0.928 (0.090)	0.952	0.893–1.000	47.0
≥RM10,000	128	0.934 (0.097)	1.000	0.919–1.000	50.9
*Self-rated health*					
Bad	7	0.927 (0.095)	1.000	0.847–1.000	52.9
Fair	248	0.855 (0.123)	0.871	0.790–0.928	20.4
Good	656	0.932 (0.093)	0.936	0.919–1.000	47.7
Very good	215	0.955 (0.078)	1.000	0.928–1.000	63.6

LSD = standard deviation; IQR = interquartile range; RM = Malaysian Ringgit. Ceiling (%) = proportion scoring 1.000 (full health). All estimates weighted to the 2020 Malaysian Census adult (≥ 18 years) population. EQ-5D-5L index scored using the Malaysian value set (Shafie et al., [29]).

**Fig. 2:**
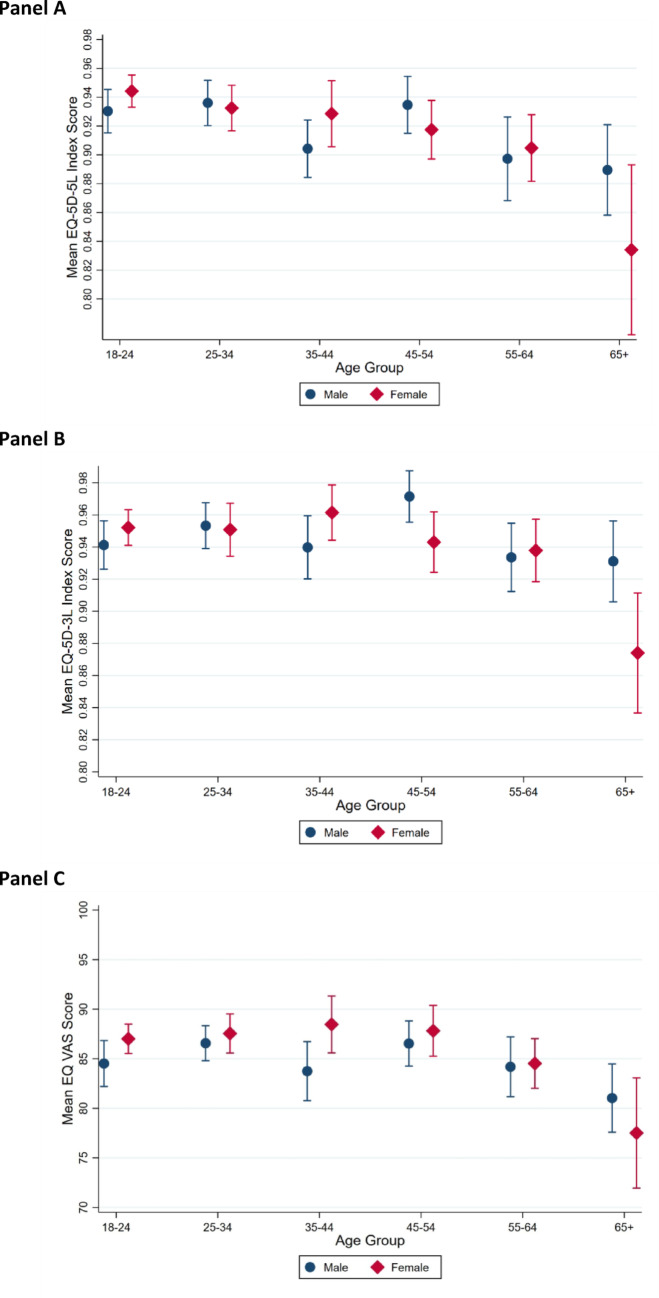
Mean EQ-5D-5L index score **A**, EQ-5D-3L index score **B**, and EQ VAS score **C** by age group and sex. Estimates are weighted to the 2020 census population. Error bars indicate 95% confidence intervals. Panels A and B enable direct comparison of age-sex patterns between the EQ-5D-5L and EQ-5D-3L.

Weighted mean EQ-5D-5L index scores varied by age, with the lowest values observed in participants aged ≥ 65 years (0.868; SD = 0.149), compared with 0.938 (SD = 0.081) in those aged 18–24 years (Fig. 2A). Males and females reported similar weighted mean scores (0.918 vs. 0.919). Chinese respondents reported the highest weighted mean index (0.928), followed by Malay (0.916) and Indian (0.910) respondents. Urban residents reported higher weighted mean scores (0.924) than rural residents (0.903) (Table 2).

### EQ-5D-3L population norms (2020 census-weighted)

EQ-5D-3L dimension data were available for 1,136 respondents, all of whom had complete data for index score computation. The weighted mean EQ-5D-3L index score was 0.945 (SD = 0.090), with a median of 1.000. The weighted ceiling effect was substantially higher at 68.5%, and only 29 unique profiles were observed (Table 3). Pain/discomfort remained the dimension with the highest prevalence of reported problems (19.4%), followed by anxiety/depression (15.3%), mobility (7.5%), usual activities (5.6%), and self-care (1.0%). Weighted mean EQ-5D-3L index scores were highest in the 45–54 age group (0.959) and lowest in those aged ≥ 65 years (0.909, SD = 0.107) (Fig. 2B). Males reported marginally higher scores than females (0.947 vs. 0.943). Indian respondents reported the lowest mean 3L index (0.922), compared with Chinese (0.948) and Malay (0.946) respondents.

**Table 3:** Census-weighted EQ-5D-3L population norms by sociodemographic characteristics (2020 Census)

Characteristic	*n*	Mean (SD)	Median	IQR	Ceiling (%)
Total	1,136	0.945 (0.090)	1.000	0.879–1.000	68.5
*Age group,years*					
18–24	304	0.947 (0.081)	1.000	0.879–1.000	67.5
25–34	255	0.952 (0.088)	1.000	0.879–1.000	72.8
35–44	165	0.949 (0.089)	1.000	0.879–1.000	71.9
45–54	168	0.959 (0.082)	1.000	1.000–1.000	75.4
55–64	152	0.936 (0.090)	1.000	0.852–1.000	62.1
≥65	92	0.909 (0.107)	1.000	0.849–1.000	50.8
*Sex*					
Male	583	0.947 (0.090)	1.000	0.879–1.000	69.9
Female	553	0.943 (0.088)	1.000	0.879–1.000	66.8
*Ethnicity*					
Malay	771	0.946 (0.087)	1.000	0.879–1.000	68.7
Chinese	288	0.948 (0.085)	1.000	0.879–1.000	69.9
Indian	67	0.922 (0.124)	1.000	0.852–1.000	62.5
Other	10	0.935 (0.090)	1.000	0.852–1.000	62.7
*Area*					
Urban	799	0.949 (0.083)	1.000	0.879–1.000	69.7
Rural	337	0.933 (0.106)	1.000	0.879–1.000	65.0
*Education*					
Primary or below	68	0.909 (0.109)	1.000	0.849–1.000	52.6
Secondary	406	0.942 (0.097)	1.000	0.879–1.000	68.5
Tertiary	662	0.951 (0.080)	1.000	0.879–1.000	70.4
*Employment*					
Employed	646	0.955 (0.083)	1.000	0.879–1.000	73.8
Student	230	0.944 (0.083)	1.000	0.852–1.000	66.4
Retired	117	0.916 (0.104)	1.000	0.852–1.000	53.8
Unemployed/other	134	0.921 (0.106)	1.000	0.879–1.000	57.3
*Marital status*					
Married/cohabiting	589	0.952 (0.083)	1.000	0.879–1.000	71.5
Never married	454	0.940 (0.095)	1.000	0.879–1.000	66.2
Widowed/divorced/separated	82	0.917 (0.109)	1.000	0.852–1.000	56.9
*Disease*					
No disease	806	0.960 (0.076)	1.000	1.000–1.000	75.5
Has disease	319	0.912 (0.107)	1.000	0.849–1.000	53.2
*Household income*					
< RM3,000	479	0.940 (0.091)	1.000	0.879–1.000	66.2
RM3,000–5,999	374	0.947 (0.090)	1.000	0.879–1.000	70.1
RM6,000–9,999	146	0.957 (0.075)	1.000	0.879–1.000	72.6
≥RM10,000	128	0.944 (0.096)	1.000	0.879–1.000	68.1
*Self-rated health*					
Bad	7	0.908 (0.111)	1.000	0.798–1.000	52.9
Fair	247	0.887 (0.112)	0.879	0.798–1.000	41.7
Good	656	0.959 (0.073)	1.000	0.879–1.000	73.7
Very good	215	0.975 (0.073)	1.000	1.000–1.000	86.2

SD = standard deviation; IQR = interquartile range; RM = Malaysian Ringgit. Ceiling (%) = proportion scoring 1.000 (full health). All estimates weighted to the 2020 Malaysian Census adult (≥ 18 years) population. EQ-5D-3L index scored using the Malaysian VAS value set (Yusof et al., [32]).

### EQ VAS (2020 census-weighted)

The weighted mean EQ VAS score was 85.4 (SD = 12.5). Scores varied with age, from 85.9 in the 18–24 age group to 79.7 among those aged ≥ 65 years, with the highest scores observed in the 45–54 age group (87.1) (Fig. 2C). Females reported slightly higher weighted mean EQ VAS (86.3) than males (84.7). Urban residents reported higher EQ VAS (86.0) than rural residents (84.4) (Table 4).

**Table 4:** Census-weighted EQ VAS population norms by sociodemographic characteristics (2020 Census)

Characteristic	*n*	Mean (SD)	Median	IQR
Total	1,137	85.4 (12.5)	90.0	80.0–95.0
*Age group, years*				
18–24	304	85.9 (11.7)	90.0	80.0–95.0
25–34	255	87.0 (10.7)	90.0	80.0–95.0
35–44	166	85.7 (14.0)	90.0	80.0–95.0
45–54	168	87.1 (11.3)	90.0	80.0–95.0
55–64	152	84.4 (12.2)	90.0	80.0–91.0
≥65	92	79.7 (14.9)	80.0	70.0–90.0
*Sex*				
Male	584	84.7 (12.8)	90.0	80.0–95.0
Female	553	86.3 (12.2)	90.0	80.0–95.0
*Ethnicity*				
Malay	772	86.5 (12.1)	90.0	80.0–95.0
Chinese	288	82.7 (13.0)	85.0	75.0–90.0
Indian	67	83.8 (14.2)	90.0	75.0–95.0
Other	10	84.2 (11.6)	90.0	70.0–90.0
*Area*				
Urban	799	85.5 (12.3)	90.0	80.0–95.0
Rural	338	85.2 (13.2)	90.0	80.0–95.0
*Education*				
Primary or below	68	80.2 (17.3)	80.0	70.0–95.0
Secondary	407	84.4 (13.8)	90.0	80.0–95.0
Tertiary	662	86.8 (10.7)	90.0	80.0–95.0
*Employment*				
Employed	647	86.2 (12.2)	90.0	80.0–95.0
Student	230	87.2 (11.3)	90.0	80.0–95.0
Retired	117	81.5 (13.0)	80.0	70.0–90.0
Unemployed/other	134	82.9 (14.3)	90.0	80.0–90.0
*Marital status*				
Married/cohabiting	589	85.8 (12.4)	90.0	80.0–95.0
Never married	455	85.0 (12.1)	90.0	80.0–95.0
Widowed/divorced/separated	82	84.2 (15.0)	90.0	80.0–95.0
*Disease*				
No disease	807	87.3 (11.0)	90.0	80.0–95.0
Has disease	319	81.1 (14.5)	80.0	70.0–90.0
*Household income*				
< RM3,000	479	85.0 (12.7)	90.0	80.0–95.0
RM3,000–5,999	375	85.7 (13.0)	90.0	80.0–95.0
RM6,000–9,999	146	86.3 (11.1)	90.0	80.0–95.0
≥RM10,000	128	85.1 (12.1)	90.0	80.0–95.0
*Self-rated health*				
Bad	7	68.7 (22.5)	60.0	50.0–90.0
Fair	248	76.1 (14.8)	80.0	70.0–90.0
Good	656	86.8 (10.2)	90.0	80.0–91.0
Very good	215	93.1 (7.2)	95.0	90.0–100.0

SD = standard deviation; IQR = interquartile range; EQ VAS = EQ visual analogue scale (0–100). All estimates weighted to the 2020 Malaysian Census adult (≥ 18 years) population.

### Comparison of EQ-5D-5L and EQ-5D-3L

The ceiling effect was reduced by 24.1% points when moving from the EQ-5D-3L to the EQ-5D-5L (68.5% vs. 44.4%, 2020-weighted). The EQ-5D-5L captured more than three times as many unique health profiles (96 vs. 29). The proportion reporting any problem was consistently higher across all five dimensions for the EQ-5D-5L (Fig. 1; Table 5). Mean EQ-5D-5L index scores were lower than mean EQ-5D-3L index scores (0.919 vs. 0.945).

**Table 5:** Head-to-head comparison of EQ-5D-5L and EQ-5D-3L (2020 census-weighted)

	EQ-5D-5L	EQ-5D-3L	Difference
*Summary scores*			
Mean index (SD)	0.919 (0.105)	0.945 (0.090)	−0.026
EQ VAS, mean (SD)	85.4 (12.5)	85.4 (12.5)	—
Ceiling effect (%)	44.4	68.5	−24.1 pp
Unique health profiles	96	29	
*% Reporting any problem*			
Mobility	17.3	7.5	9.8 pp
Self-care	4.0	1.0	3.0 pp
Usual activities	14.9	5.6	9.4 pp
Pain/discomfort	36.0	19.4	16.6 pp
Anxiety/depression	28.4	15.3	13.1 pp

SD = standard deviation; pp = percentage points; EQ VAS = EQ visual analogue scale. EQ-5D-5L index scored using the Malaysian DCE-TTO hybrid value set (Shafie et al., 20 [29] EQ-5D-3L index scored using the Malaysian VAS value set (Yusof et al., [32]).

### Multivariable regression analysis

Results from the multivariable regression analyses are presented in Table 6. In the OLS model for the EQ-5D-5L index (*n* = 1,126; R² = 0.107), having a doctor-diagnosed disease was the strongest predictor of lower health status (β = −0.042, 95% CI − 0.057, − 0.027), followed by being in the 65 + age group (β = −0.049, 95% CI − 0.088, − 0.009) and the 35–44 age group (β = −0.030, 95% CI − 0.054, − 0.007), relative to the 18–24 reference category. Compared with those who were employed, being unemployed or outside the labour force was also associated with lower index scores (β = −0.023, 95% CI − 0.045 to − 0.001).

**Table 6:** Multivariable regression results: factors associated with EQ-5D-5L index, EQ-5D-3L index, and EQ VAS

	OLS 5L		Tobit 5L		OLS 3L		Tobit 3L		OLS VAS		GLM VAS
	β (95% CI)		β (95% CI)		β (95% CI)		β (95% CI)		β (95% CI)		β (95% CI)
*Sex (Ref: male)*											
Female	0.004 (− 0.008, 0.017)		0.007 (− 0.014, 0.029)		0.004 (− 0.007, 0.015)		0.005 (− 0.028, 0.038)		2.01 (0.51, 3.50) ^**^		0.024 (0.007, 0.042) ^**^
*Residence (Ref: urban)*											
Rural	−0.013 (− 0.028, 0.001)		−0.022 (− 0.045, 0.001)		−0.014 (− 0.026, − 0.002) ^*^		−0.035 (− 0.070, 0.000)		−0.06 (− 1.69, 1.58)		−0.001 (− 0.020, 0.019)
*Disease (Ref: no)*											
Has disease	−0.042 (− 0.057, − 0.027) ^***^		−0.071 (− 0.095, − 0.048) ^***^		−0.040 (− 0.052, − 0.027) ^***^		−0.107 (− 0.143, − 0.072) ^***^		−5.23 (− 7.02, − 3.45) ^***^		−0.062 (− 0.084, − 0.041) ^***^
*Age (Ref: 18–24)*											
25–34	−0.015 (− 0.034, 0.003)		−0.027 (− 0.063, 0.009)		−0.009 (− 0.027, 0.009)		−0.009 (− 0.064, 0.046)		0.22 (− 2.06, 2.50)		0.001 (− 0.025, 0.028)
35–44	−0.030 (− 0.054, − 0.007) ^*^		−0.051 (− 0.095, − 0.006) ^*^		−0.013 (− 0.035, 0.010)		−0.017 (− 0.086, 0.052)		−0.42 (− 3.60, 2.76)		−0.007 (− 0.044, 0.031)
45–54	−0.018 (− 0.042, 0.006)		−0.027 (− 0.074, 0.020)		0.000 (− 0.022, 0.023)		0.016 (− 0.057, 0.088)		1.37 (− 1.77, 4.51)		0.016 (− 0.021, 0.053)
55–64	−0.032 (− 0.064, − 0.001) ^*^		−0.050 (− 0.102, 0.002)		−0.006 (− 0.032, 0.019)		−0.019 (− 0.098, 0.060)		0.28 (− 3.65, 4.20)		0.004 (− 0.043, 0.050)
65+	−0.049 (− 0.088, − 0.009) ^*^		−0.076 (− 0.136, − 0.015) ^*^		−0.024 (− 0.055, 0.007)		−0.054 (− 0.146, 0.038)		−2.27 (− 6.85, 2.31)		−0.028 (− 0.083, 0.028)
*Ethnicity (Ref: malay)*											
Chinese	0.013 (− 0.001, 0.026)		0.031 (0.006, 0.055) ^*^		0.004 (− 0.007, 0.016)		0.019 (− 0.019, 0.057)		−3.68 (− 5.39, − 1.96) ^***^		−0.043 (− 0.064, − 0.023) ^***^
Indian	−0.011 (− 0.040, 0.017)		−0.010 (− 0.053, 0.032)		−0.029 (− 0.059, 0.001)		−0.057 (− 0.119, 0.006)		−3.40 (− 6.86, 0.06)		−0.041 (− 0.083, 0.001)
Other	0.023 (− 0.033, 0.080)		0.078 (− 0.041, 0.197)		−0.001 (− 0.049, 0.046)		−0.004 (− 0.169, 0.161)		−1.85 (− 8.33, 4.64)		−0.021 (− 0.098, 0.055)
*Education (Ref: tertiary)*											
Primary or below	−0.023 (− 0.057, 0.012)		−0.024 (− 0.075, 0.028)		−0.012 (− 0.040, 0.016)		−0.017 (− 0.095, 0.061)		−3.99 (− 8.25, 0.27)		−0.051 (− 0.103, 0.001)
Secondary	−0.013 (− 0.028, 0.002)		−0.019 (− 0.045, 0.008)		−0.004 (− 0.019, 0.011)		−0.006 (− 0.047, 0.034)		−1.96 (− 3.93, 0.02)		−0.024 (− 0.047, 0.000)
*Employment (Ref: employed)*											
Student	−0.015 (− 0.031, 0.001)		−0.025 (− 0.061, 0.010)		−0.015 (− 0.032, 0.002)		−0.044 (− 0.097, 0.009)		0.54 (− 1.70, 2.78)		0.006 (− 0.020, 0.032)
Retired	−0.019 (− 0.048, 0.010)		−0.028 (− 0.069, 0.014)		−0.022 (− 0.045, 0.001)		−0.057 (− 0.120, 0.005)		−1.71 (− 4.87, 1.44)		−0.021 (− 0.059, 0.017)
Unemployed/other	−0.023 (− 0.045, − 0.001) ^*^		−0.038 (− 0.072, − 0.004) ^*^		−0.026 (− 0.045, − 0.007) ^**^		−0.066 (− 0.117, − 0.014) ^*^		−2.04 (− 4.55, 0.47)		−0.025 (− 0.055, 0.005)
*Marital (Ref: married)*											
Never married	−0.017 (− 0.036, 0.001)		−0.030 (− 0.061, 0.002)		−0.022 (− 0.039, − 0.004) ^*^		−0.054 (− 0.102, − 0.006) ^*^		−2.88 (− 5.13, − 0.62) ^*^		−0.035 (− 0.062, − 0.008) ^*^
Widowed/div./sep.	0.002 (− 0.029, 0.033)		−0.002 (− 0.043, 0.039)		−0.024 (− 0.048, 0.001)		−0.053 (− 0.114, 0.007)		−0.45 (− 3.87, 2.96)		−0.006 (− 0.047, 0.036)
*Income (Ref: < RM3,000)*											
RM3,000–5,999	0.006 (− 0.008, 0.021)		0.013 (− 0.011, 0.038)		−0.009 (− 0.022, 0.003)		−0.025 (− 0.063, 0.012)		−0.41 (− 2.13, 1.31)		−0.005 (− 0.025, 0.015)
RM6,000–9,999	0.003 (− 0.015, 0.021)		0.004 (− 0.030, 0.039)		0.001 (− 0.015, 0.016)		0.001 (− 0.053, 0.054)		−0.80 (− 3.00, 1.41)		−0.009 (− 0.035, 0.017)
≥RM10,000	0.007 (− 0.012, 0.027)		0.016 (− 0.020, 0.053)		−0.014 (− 0.034, 0.006)		−0.035 (− 0.090, 0.020)		−1.69 (− 4.38, 0.99)		−0.020 (− 0.053, 0.012)
N	1,126		1,126		1,125		1,125		1,126		1,126
R²	0.107		—		0.096		—		0.095		—
Pseudo R²	—		0.394		—		0.122		—		—

*p < 0.05, **p < 0.01, ***p < 0.001. β = regression coefficient; CI = confidence interval; OLS = ordinary least squares; GLM = generalised linear model; VAS = visual analogue scale; RM = Malaysian Ringgit. OLS models use HC1 robust standard errors. Tobit models are upper-censored at 1.0. GLM uses Gamma family with log-link and robust standard errors.

The Tobit model, which accounted for the substantial proportion (45.4% unweighted; 44.4% weighted) of observations censored at the upper bound of 1.0, produced broadly similar results, although with larger coefficient magnitudes, as expected. Having a diagnosed disease (β = −0.071, 95% CI − 0.095 to − 0.048), being aged ≥ 65 years (β = −0.077, 95% CI − 0.136 to − 0.015), and being unemployed (β = −0.040, 95% CI − 0.072 to − 0.004) remained strongly associated with lower latent health utility. Chinese ethnicity was associated with higher latent health utility (β = 0.033, 95% CI). Rural residence showed marginal significance in the Tobit model (β = −0.023, 95% CI − 0.045, 0.001).

In the OLS model for the EQ-5D-3L index (*n* = 1,125; R² = 0.096), having a diagnosed disease showed the strongest association with lower health status (β = −0.040, 95% CI − 0.052 to − 0.027). This was followed by being unemployed or outside the labour force (β = −0.026, 95% CI − 0.045 to − 0.007), never being married (β = −0.022, 95% CI − 0.039 to − 0.004), and rural residence (β = −0.014, 95% CI − 0.026 to − 0.002). In the Tobit model for the EQ-5D-3L, in which 68.2% of observations were censored at 1.0, diagnosed disease (β = −0.107, 95% CI − 0.143 to − 0.072), unemployment (β = −0.063, 95% CI − 0.117 to − 0.014), and never being married (β = −0.052, 95% CI − 0.102 to − 0.006) remained significantly associated with lower latent health utility.

In the OLS model for EQ VAS (R² = 0.095), having a diagnosed disease showed the strongest association with lower scores (β = −5.23, 95% CI − 7.02 to − 3.45). Chinese ethnicity was also associated with lower EQ VAS scores (β= −3.68, 95% CI − 5.39 to − 1.96). Being female was associated with higher EQ VAS scores (β = 2.01, 95% CI 0.51 to 3.50), whereas never being married was associated with lower scores (β = −2.88, 95% CI − 5.13 to − 0.62). The modified Park test supported a Gamma variance function for the EQ VAS, and the Pregibon link test confirmed the adequacy of the log-link specification (Supplementary Table S8). The GLM sensitivity analysis (Gamma distribution with log link) produced substantively consistent results (Table 6).

### Sensitivity analysis with 2010 census weights

Results from the sensitivity analysis using 2010 census weights were consistent with the primary analysis. The weighted mean EQ-5D-5L index score was 0.921 (SD = 0.102), and the ceiling effect was 45.4%, compared with 44.4% using 2020 weights (Supplementary Table S1). Minor differences reflect variations in age and area distributions between the 2010 and 2020 censuses, while overall patterns by age group, sex, and area of residence were unchanged. The level-specific response distributions for both instruments are provided in Supplementary Table S9. Unadjusted (unweighted) estimates are provided in Supplementary Table S10 for comparison; the unweighted EQ-5D-5L mean (0.918) was nearly identical to the weighted estimate (0.919).

## Discussion

This study presents the first population norms for both the EQ-5D-3L and EQ-5D-5L in Peninsular Malaysia, weighted to the 2020 Malaysian census population structure. The weighted mean EQ-5D-5L index score of 0.919 and mean EQ VAS of 85.4 indicate generally high self-reported health in the Malaysian adult population. Sensitivity analyses using 2010 census weights produced nearly identical estimates, with weighted and unweighted means differing by less than 0.003, supporting the robustness of the norms across alternative weighting approaches. These data address an important gap in the Malaysian HRQoL literature and provide reference values for clinical research, economic evaluation, and health policy.

The Malaysian EQ-5D-5L mean index score is comparable with estimates from other Southeast Asian countries, including Thailand (0.92 using the Thai value set) [26], Indonesia (0.91) [22], Hong Kong (0.92) [25], Singapore (0.94) [24], and Japan (0.92) [20]. The mean EQ VAS score of 85.4 exceeds the pooled mean of 79.0 reported across 23 countries by Wang et al. [19]. The EQ-5D-5L ceiling effect of 44.4% (2020-weighted) was similar to levels observed in Asian settings, such as Japan (55%) [20] and Thailand (49%) [26]. These comparisons should be interpreted cautiously because differences in value sets, sampling strategies, population characteristics, and age structure can influence estimated norms [19, 41, 42]. Nonethless, the observed pattern may reflect cultural differences in health reporting or genuinely better self-reported health among younger and healthier Asian populations.

Pain/discomfort and anxiety/depression were the most frequently reported problems on both instruments [14, 19, 27]. On the EQ-5D-5L, 36.0% of respondents reported pain or discomfort and 28.4% reported anxiety or depression (Supplementary Table S6). These findings align with international evidence showing pain/discomfort as the most prevalent EQ-5D dimension and self-care as the least affected [19]. The relatively high prevalence of anxiety/depression in this sample merits attention and may be linked to urbanisation, economic pressures, and social change in Malaysia [43]. Self-care problems were uncommon (4.0%), consistent with the relatively young sample and high levels of functional independence.

Health status varied systematically across demographic groups (Supplementary Tables S3–S5; Supplementary Fig. S2). EQ-5D-5L index scores were lower in older age groups, ranging from 0.938 among those aged 18–24 years to 0.868 among those aged ≥ 65 years, reflecting the accumulation of chronic conditions and functional limitations with age [14, 19, 27]. Scores were similar between males and females, contrasting with patterns reported in many Western studies but aligning with findings from Singapore [24, 44]. Rural residents reported poorer health than urban residents, consistent with disparities in healthcare access, socioeconomic conditions, and occupational risks in rural Malaysia.

Regression analyses provided further insight into factors independently associated with HRQoL. Across all models, having a doctor-diagnosed disease showed the strongest association with lower health status, highlighting the central role of chronic disease burden [45]. Tobit models, which accounted for ceiling effects, produced larger coefficient magnitudes than OLS models, consistent with attenuation in the presence of censoring [37, 38]. Chinese ethnicity was associated with higher EQ-5D-5L index scores but lower EQ VAS scores relative to Malay respondents, a pattern also observed in other multi-ethnic Asian populations [24, 44]. This divergence may reflect differences in the interpretation of numerical rating scales compared with categorical health descriptions. In the EQ-5D-3L models, unemployment, never married status, and rural residence were also associated with poorer health. The consistency of key correlates across instruments, particularly diagnosed disease, employment status, and marital status, supports the robustness of these findings. Sex differences varied by outcome, with female sex associated with higher EQ VAS scores but not with EQ-5D-5L index scores, suggesting instrument-specific reporting patterns. These findings suggest that the EQ-5D descriptive system and EQ VAS capture related but distinct constructs; the EQ VAS may be more sensitive to broader psychosocial factors that influence holistic health assessments, whereas the EQ-5D index reflects categorical responses scored through a population value set. Future research should examine response style patterns and cultural influences on health reporting in multi-ethnic populations.

Direct comparison of the EQ-5D-3L and EQ-5D-5L confirmed the superior measurement performance of the 5L version. The marked reduction in ceiling effects (from 68.5% to 44.4%) and the increase in unique health profiles (from 29 to 96) demonstrate its improved discriminatory capacity. These findings are consistent with international evidence [10, 11] and previous Malaysian validation work [30]. Greater sensitivity to mild and moderate health problems makes the EQ-5D-5L more suitable for general population surveys and clinical studies [10]. Tobit analyses also indicate that variation in population health is greater than that reflected by observed EQ-5D index scores. The EQ-5D-5L value set was derived using a C-TTO/DCE hybrid model, whereas the EQ-5D-3L value set was VAS-based. These methodological differences in valuation approach should be considered when comparing absolute index scores between the two instruments, as VAS-based value sets tend to produce a more compressed range. Notably, age effects were more pronounced in the EQ-5D-5L regression models, with age groups 35–44, 55–64, and 65 + reaching significance, whereas none reached significance for the EQ-5D-3L. This likely reflects the superior discriminatory power of the 5L version, as the higher ceiling effect of the 3L compresses variation and attenuates regression coefficients.

### Applications of the population norms

The population norms generated in this study have several practical applications. First, they provide benchmarks for burden-of-disease studies, allowing patient groups to be compared with age- and sex-matched population values. For example, a Malaysian cancer study reported a mean EQ-5D-5L index of 0.76 [46], indicating a substantial health decrement relative to the population norm. Second, the norms support QALY estimation in economic evaluations. The Malaysian Pharmacoeconomic Guidelines recommend the use of locally derived utility values [28], and these norms can serve as baseline utilities in cost-effectiveness models. This is particularly relevant as pharmacoeconomic evidence increasingly informs formulary and reimbursement decisions in Malaysia [47]. Third, the stratified norms inform health policy and resource allocation. Urban–rural differences highlight areas for targeted intervention, while dimension-level findings emphasise the need to prioritise pain management and mental health services. Fourth, availability of norms for both EQ-5D versions facilitates the transition from the 3L to the 5L, supports mapping between instruments [48], and enables continuity in longitudinal research. Finally, these norms contribute to international comparisons of population health, supporting regional benchmarking and policy dialogue within ASEAN and the broader global health community.

### Strengths and limitations

This study has several strengths. Quota-based sampling across four regional clusters, with both urban and rural representation, supports population representativeness. Face-to-face, interviewer-administered data collection using the standardised EQ-VT protocol [12] ensured data quality. Administering the EQ-5D-3L and EQ-5D-5L to the same respondents enabled direct comparison, while the large sample size (*n* = 1,137), high completion rate (97.0%), and census-weighted analyses with sensitivity testing further strengthen the robustness of the norms.

Several limitations should be considered. The study was restricted to Peninsular Malaysia, and findings may not generalise to East Malaysia (Sabah and Sarawak). Minor demographic imbalances required weighting, although the small design effect (DEFF = 1.125) indicates minimal efficiency loss (Supplementary Table S7). The cross-sectional design limits causal inference. Data collected in 2016 provide a pre-pandemic baseline; rather than reducing relevance, this strengthens the value of the norms for assessing post-pandemic changes in population health, particularly for anxiety/depression and pain/discomfort. The fixed order of instrument administration may have introduced a sequence effect [10]; however, high dimension-level correlations between instruments and consistency with international patterns suggest this effect was minimal. Randomisation of instrument order was not feasible within the standardised EQ-VT valuation protocol. Furthermore, the population norms were derived from the same sample used for the Malaysian EQ-5D-5L valuation study; however, self-reported health data were collected before any valuation tasks, minimising the risk of context effects. Dedicated population surveys such as those in the EQ-DAPHNE programme offer advantages including larger, independent samples [49], although online panel-based designs may under-represent populations with limited internet access. Future studies should collect norms from an independent, larger sample, including East Malaysia. Some demographic strata (e.g. Other ethnicity, *n* = 10) have small sample sizes, and their estimates should be interpreted with caution. Finally, the EQ-5D-3L value set was VAS-based [32], which should be considered when making international comparisons.

## Conclusion

This study establishes the first population norms for the EQ-5D-3L and EQ-5D-5L in Peninsular Malaysia using 2020 census weights. The weighted mean EQ-5D-5L index score was 0.919 and the mean EQ VAS was 85.4, indicating generally good self-reported health, while the mean EQ-5D-3L index score was 0.945. Estimates were consistent across census weighting approaches. Health status was lower among older adults, individuals with diagnosed diseases, and rural residents, with pain/discomfort and anxiety/depression reported most frequently. Across regression models, diagnosed disease, older age, and unemployment showed consistent associations with poorer health. Compared with the EQ-5D-3L, the EQ-5D-5L demonstrated superior discriminatory performance, reflected by a substantially lower ceiling effect. These norms provide robust reference data for economic evaluation, health policy, and equity-focused analyses in Malaysia.

## Supplementary Information

Below is the link to the electronic supplementary material.

Supplementary Material 1

## Data Availability

The dataset analysed during the current study is available from the corresponding author upon reasonable request.
