# Supplementary material for: Population norms for the EQ-5D-3L and EQ-5D-5L in Peninsular Malaysia
Source: Qual Life Res. 2026 Apr 1;35(5):122. doi: 10.1007/s11136-026-04231-x (PMC13043599; doi:10.1007/s11136-026-04231-x)
Supplement: Supplementary file 1 — Supplementary Material 1 [file 11136_2026_4231_MOESM1_ESM.docx]

**Online Resource 1: Supplementary Tables and Figures**

**Table S1 Sensitivity analysis: EQ-5D-5L population norms using 2010 census weights**

| **Characteristic** | **n** | **Mean (SD)** | **Ceiling (%)** |
| --- | --- | --- | --- |
| **Total** | 1,137 | 0.921 (0.102) | 45.4 |
| ***Age group, years*** |  |  |  |
| 18–24 | 304 | 0.939 (0.081) | 50.6 |
| 25–34 | 255 | 0.934 (0.091) | 48.6 |
| 35–44 | 166 | 0.915 (0.101) | 42.3 |
| 45–54 | 168 | 0.924 (0.096) | 48.0 |
| 55–64 | 152 | 0.899 (0.117) | 40.8 |
| ≥65 | 92 | 0.868 (0.152) | 28.4 |
| ***Sex*** |  |  |  |
| Male | 584 | 0.920 (0.102) | 45.3 |
| Female | 553 | 0.923 (0.102) | 45.5 |
| ***Area*** |  |  |  |
| Urban | 799 | 0.927 (0.096) | 47.3 |
| Rural | 338 | 0.908 (0.114) | 40.6 |

*SD = standard deviation. Ceiling (%) = proportion scoring 1.000 (full health). All estimates weighted to the 2010 Malaysian Census adult (≥18 years) population.*

**Table S2 Most frequently reported EQ-5D health profiles**

| **5L Profile** | **n** | **%** |  | **3L Profile** | **n** | **%** |
| --- | --- | --- | --- | --- | --- | --- |
| 11111 | 516 | 45.4 |  | 11111 | 774 | 68.1 |
| 11121 | 128 | 11.3 |  | 11121 | 105 | 9.2 |
| 11112 | 126 | 11.1 |  | 11112 | 93 | 8.2 |
| 11122 | 55 | 4.8 |  | 11122 | 41 | 3.6 |
| 21121 | 34 | 3.0 |  | 21121 | 20 | 1.8 |
| 21111 | 21 | 1.8 |  | 21111 | 17 | 1.5 |
| 11221 | 16 | 1.4 |  | 11211 | 15 | 1.3 |
| 11211 | 16 | 1.4 |  | 21221 | 13 | 1.1 |
| 11222 | 16 | 1.4 |  | 21122 | 11 | 1.0 |
| 21122 | 13 | 1.1 |  | 11222 | 8 | 0.7 |
| 31121 | 13 | 1.1 |  | 11221 | 8 | 0.7 |
| 11212 | 12 | 1.1 |  | 21222 | 6 | 0.5 |
| 21221 | 10 | 0.9 |  | 11212 | 4 | 0.4 |
| 21222 | 9 | 0.8 |  | 21112 | 4 | 0.4 |
| 11223 | 9 | 0.8 |  | 21212 | 3 | 0.3 |

*Total unique profiles: EQ-5D-5L = 96; EQ-5D-3L = 29. Profile digits represent responses to mobility, self-care, usual activities, pain/discomfort, and anxiety/depression dimensions respectively (1 = no problems, 5 = extreme problems for EQ-5D-5L; 1 = no problems, 3 = extreme problems for EQ-5D-3L).*

**Table S3 Cross-tabulated population norms by age group and sex (2020 census-weighted)**

***Panel A: EQ-5D-5L index***

| **Age group** | **Male, mean (SD)** | **n** | **Female, mean (SD)** | **n** |
| --- | --- | --- | --- | --- |
| 18–24 | 0.930 (0.087) | 128 | 0.944 (0.076) | 176 |
| 25–34 | 0.936 (0.097) | 149 | 0.933 (0.083) | 106 |
| 35–44 | 0.904 (0.099) | 94 | 0.929 (0.099) | 72 |
| 45–54 | 0.935 (0.094) | 87 | 0.917 (0.093) | 81 |
| 55–64 | 0.897 (0.126) | 72 | 0.905 (0.105) | 80 |
| ≥65 | 0.890 (0.118) | 54 | 0.834 (0.186) | 38 |

***Panel B: EQ-5D-3L index***

| **Age group** | **Male, mean (SD)** | **n** | **Female, mean (SD)** | **n** |
| --- | --- | --- | --- | --- |
| 18–24 | 0.941 (0.087) | 128 | 0.952 (0.075) | 176 |
| 25–34 | 0.953 (0.089) | 149 | 0.951 (0.087) | 106 |
| 35–44 | 0.940 (0.097) | 93 | 0.962 (0.075) | 72 |
| 45–54 | 0.972 (0.076) | 87 | 0.943 (0.086) | 81 |
| 55–64 | 0.934 (0.092) | 72 | 0.938 (0.089) | 80 |
| ≥65 | 0.931 (0.095) | 54 | 0.874 (0.118) | 38 |

***Panel C: EQ VAS***

| **Age group** | **Male, mean (SD)** | **n** | **Female, mean (SD)** | **n** |
| --- | --- | --- | --- | --- |
| 18–24 | 84.5 (13.4) | 128 | 87.0 (10.0) | 176 |
| 25–34 | 86.6 (11.0) | 149 | 87.6 (10.4) | 106 |
| 35–44 | 83.7 (14.7) | 94 | 88.5 (12.4) | 72 |
| 45–54 | 86.5 (10.8) | 87 | 87.8 (11.8) | 81 |
| 55–64 | 84.2 (13.1) | 72 | 84.5 (11.4) | 80 |
| ≥65 | 81.0 (12.9) | 54 | 77.5 (17.5) | 38 |

*SD = standard deviation; EQ VAS = EQ visual analogue scale. All estimates weighted to the 2020 Malaysian Census. Cells with n < 10 are suppressed (—).*

**Table S4 Cross-tabulated population norms by ethnicity and sex (2020 census-weighted)**

***Panel A: EQ-5D-5L index***

| **Ethnicity** | **Male, mean (SD)** | **n** | **Female, mean (SD)** | **n** |
| --- | --- | --- | --- | --- |
| Malay | 0.918 (0.096) | 399 | 0.914 (0.113) | 373 |
| Chinese | 0.925 (0.108) | 139 | 0.930 (0.092) | 149 |
| Indian | 0.897 (0.143) | 41 | 0.931 (0.079) | 26 |
| Other | — | — | — | — |

***Panel B: EQ-5D-3L index***

| **Ethnicity** | **Male, mean (SD)** | **n** | **Female, mean (SD)** | **n** |
| --- | --- | --- | --- | --- |
| Malay | 0.951 (0.083) | 398 | 0.940 (0.091) | 373 |
| Chinese | 0.946 (0.090) | 139 | 0.951 (0.079) | 149 |
| Indian | 0.910 (0.137) | 41 | 0.943 (0.098) | 26 |
| Other | — | — | — | — |

***Panel C: EQ VAS***

| **Ethnicity** | **Male, mean (SD)** | **n** | **Female, mean (SD)** | **n** |
| --- | --- | --- | --- | --- |
| Malay | 86.1 (12.1) | 399 | 87.0 (12.1) | 373 |
| Chinese | 81.7 (13.5) | 139 | 83.9 (12.4) | 149 |
| Indian | 80.6 (14.9) | 41 | 89.1 (11.4) | 26 |
| Other | — | — | — | — |

*SD = standard deviation; EQ VAS = EQ visual analogue scale. Cells with n < 10 are suppressed (—).*

**Table S5 Cross-tabulated population norms by age group and ethnicity (2020 census-weighted)**

***Panel A: EQ-5D-5L index***

| **Age group** | **Malay** | **n** | **Chinese** | **n** | **Indian** | **n** |
| --- | --- | --- | --- | --- | --- | --- |
| 18–24 | 0.934 (0.082) | 201 | 0.952 (0.075) | 87 | 0.928 (0.094) | 15 |
| 25–34 | 0.942 (0.073) | 184 | 0.924 (0.114) | 45 | 0.886 (0.162) | 21 |
| 35–44 | 0.914 (0.100) | 116 | 0.907 (0.105) | 41 | — | — |
| 45–54 | 0.922 (0.096) | 119 | 0.942 (0.085) | 39 | — | — |
| 55–64 | 0.885 (0.123) | 93 | 0.955 (0.058) | 45 | 0.854 (0.139) | 12 |
| ≥65 | 0.851 (0.157) | 59 | 0.894 (0.133) | 31 | — | — |

***Panel B: EQ-5D-3L index***

| **Age group** | **Malay** | **n** | **Chinese** | **n** | **Indian** | **n** |
| --- | --- | --- | --- | --- | --- | --- |
| 18–24 | 0.944 (0.081) | 201 | 0.960 (0.071) | 87 | 0.930 (0.106) | 15 |
| 25–34 | 0.961 (0.072) | 184 | 0.942 (0.101) | 45 | 0.895 (0.151) | 21 |
| 35–44 | 0.946 (0.092) | 116 | 0.956 (0.085) | 41 | — | — |
| 45–54 | 0.953 (0.081) | 119 | 0.978 (0.055) | 39 | — | — |
| 55–64 | 0.939 (0.092) | 93 | 0.947 (0.071) | 45 | 0.882 (0.116) | 12 |
| ≥65 | 0.910 (0.112) | 59 | 0.904 (0.100) | 31 | — | — |

***Panel C: EQ VAS***

| **Age group** | **Malay** | **n** | **Chinese** | **n** | **Indian** | **n** |
| --- | --- | --- | --- | --- | --- | --- |
| 18–24 | 88.0 (9.8) | 201 | 83.0 (12.6) | 87 | 75.5 (17.8) | 15 |
| 25–34 | 88.1 (10.0) | 184 | 80.7 (11.3) | 45 | 87.0 (12.7) | 21 |
| 35–44 | 86.8 (13.2) | 116 | 82.3 (16.1) | 41 | — | — |
| 45–54 | 87.3 (10.9) | 119 | 86.4 (11.7) | 39 | — | — |
| 55–64 | 84.7 (13.1) | 93 | 85.5 (9.4) | 45 | 78.9 (12.4) | 12 |
| ≥65 | 80.0 (16.0) | 59 | 79.1 (13.3) | 31 | — | — |

*SD = standard deviation; EQ VAS = EQ visual analogue scale. Values presented as mean (SD) n=sample size. Cells with n < 10 are suppressed (—).*

**Table S6 Proportion (%) reporting any problem by EQ-5D dimension and age group (2020 census-weighted)**

| **Age group** | **MO** | **SC** | **UA** | **PD** | **AD** |
| --- | --- | --- | --- | --- | --- |
| ***EQ-5D-5L*** |  |  |  |  |  |
| 18–24 | 5.8 | 2.6 | 13.8 | 30.2 | 27.9 |
| 25–34 | 6.8 | 2.5 | 13.2 | 27.3 | 33.1 |
| 35–44 | 16.5 | 3.5 | 16.9 | 38.3 | 32.9 |
| 45–54 | 19.0 | 2.4 | 13.1 | 35.4 | 23.5 |
| 55–64 | 31.3 | 6.0 | 15.7 | 42.5 | 25.9 |
| ≥65 | 42.2 | 10.7 | 18.1 | 52.9 | 19.6 |
| ***EQ-5D-3L*** |  |  |  |  |  |
| 18–24 | 4.0 | 0.4 | 6.8 | 19.4 | 16.2 |
| 25–34 | 4.2 | 0.4 | 5.4 | 13.0 | 17.2 |
| 35–44 | 6.0 | 1.4 | 4.4 | 16.9 | 16.5 |
| 45–54 | 4.7 | 1.0 | 3.8 | 17.9 | 9.4 |
| 55–64 | 12.4 | 0.5 | 3.7 | 24.7 | 15.5 |
| ≥65 | 21.8 | 2.9 | 10.7 | 34.8 | 15.6 |

*MO = mobility; SC = self-care; UA = usual activities; PD = pain/discomfort; AD = anxiety/depression.*

**Table S7 Survey weight diagnostics**

|  | **2020 Census weights** | **2010 Census weights** |
| --- | --- | --- |
| Mean | 1.000 | 1.000 |
| Range | 0.378–2.205 | 0.564–1.830 |
| CV | 0.353 | 0.230 |
| DEFF | 1.125 | 1.053 |

*CV = coefficient of variation; DEFF = design effect (1 + CV²).*

**Table S8 GLM specification test results for EQ VAS**

| **Test** | **Result** |
| --- | --- |
| Modified Park test coefficient | ≈2.0 (supports Gamma variance function) |
| Pregibon link test (_hatsq) | p > 0.05 (log-link adequate) |
| ***AIC comparison*** |  |
| OLS (Gaussian-identity) | 8,767.80 |
| GLM (Gamma-log) | 12,312.28 |
| Pearson dispersion statistic | 0.0197 (close to 1/df Pearson; Gamma fit adequate) |

*AIC = Akaike information criterion. The modified Park test regresses log(squared residuals) on log(predicted values); a coefficient ≈2 is consistent with a Gamma variance function. The Pregibon link test examines the significance of the squared linear predictor (_hatsq); non-significance supports the log-link. AIC values are not directly comparable across families (OLS vs GLM); however, the Gamma-log GLM was preferred based on the Park and Pregibon tests.*

**Table S9 Response distribution by dimension and response level for EQ-5D-5L and EQ-5D-3L (2020 census-weighted)**

***Panel A: EQ-5D-5L***

| **Response level** | **MO, n (%)** | **SC, n (%)** | **UA, n (%)** | **PD, n (%)** | **AD, n (%)** |
| --- | --- | --- | --- | --- | --- |
| Level 1 | 956 (82.7) | 1,094 (96.0) | 970 (85.1) | 736 (64.0) | 812 (71.6) |
| Level 2 | 128 (11.8) | 31 (3.0) | 132 (11.8) | 342 (30.7) | 271 (23.2) |
| Level 3 | 48 (4.9) | 10 (0.9) | 30 (2.7) | 54 (4.8) | 46 (4.4) |
| Level 4 | 5 (0.5) | 2 (0.2) | 4 (0.4) | 2 (0.2) | 5 (0.5) |
| Level 5 | 0 (0.0) | 0 (0.0) | 1 (0.1) | 3 (0.2) | 3 (0.3) |

***Panel B: EQ-5D-3L***

| **Response level** | **MO, n (%)** | **SC, n (%)** | **UA, n (%)** | **PD, n (%)** | **AD, n (%)** |
| --- | --- | --- | --- | --- | --- |
| Level 1 | 1,056 (92.5) | 1,126 (99.0) | 1,070 (94.4) | 914 (80.6) | 959 (84.7) |
| Level 2 | 80 (7.5) | 9 (0.9) | 65 (5.5) | 220 (19.2) | 174 (15.0) |
| Level 3 | 0 (0.0) | 1 (0.1) | 1 (0.1) | 2 (0.2) | 3 (0.3) |

*MO = mobility; SC = self-care; UA = usual activities; PD = pain/discomfort; AD = anxiety/depression. Percentages are weighted to the 2020 Malaysian Census adult (≥18 years) population. Level 1 = no problems; Level 2 = slight problems (5L) / some problems (3L); Level 3 = moderate problems (5L) / extreme problems (3L); Level 4 = severe problems (5L only); Level 5 = extreme problems (5L only).*

**Table S10 Unadjusted (unweighted) EQ-5D population norms**

| **Statistic** | **EQ-5D-5L index** | **EQ-5D-3L index** | **EQ VAS** |
| --- | --- | --- | --- |
| n | 1,137 | 1,136 | 1,137 |
| Mean (SD) | 0.921 (0.103) | 0.945 (0.089) | 85.5 (12.3) |
| Median | 0.928 | 1.000 | 90.0 |
| Ceiling (%) | 45.4 | 68.1 | — |
| Range | 0.135–1.000 | 0.324–1.000 | 30–100 |

*SD = standard deviation. Ceiling (%) = proportion scoring 1.000 (full health). All estimates are unweighted (unadjusted). The unweighted EQ-5D-5L mean (0.921) is nearly identical to the 2020 census-weighted estimate (0.919, Table 2), confirming minimal impact of post-stratification weighting.*


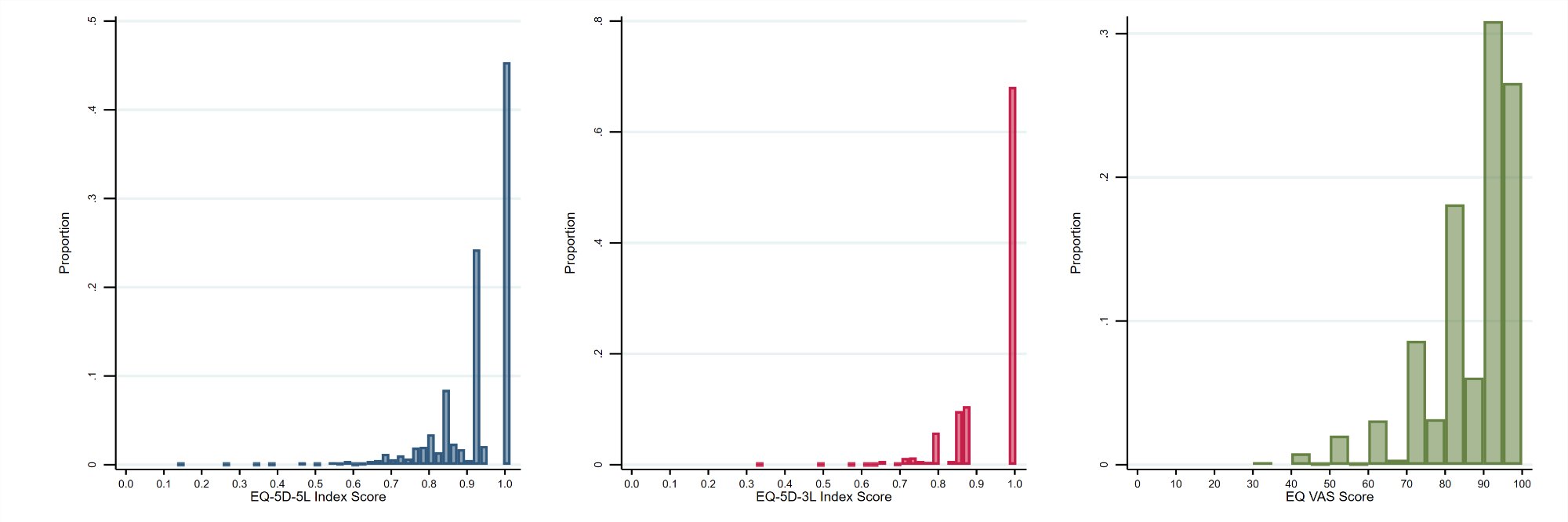


**Fig. S1 Distribution of (a) EQ-5D-5L index, (b) EQ-5D-3L index, and (c) EQ VAS scores**

*EQ-5D-5L = five-level EQ-5D; EQ-5D-3L = three-level EQ-5D; EQ VAS = EQ visual analogue scale.*


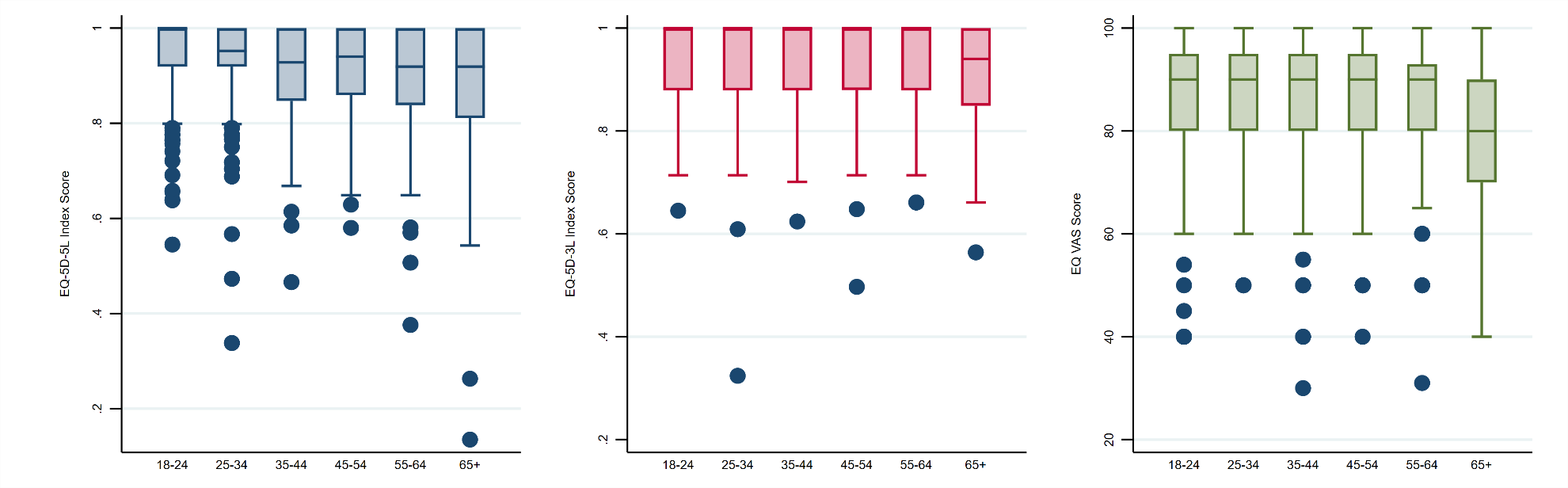


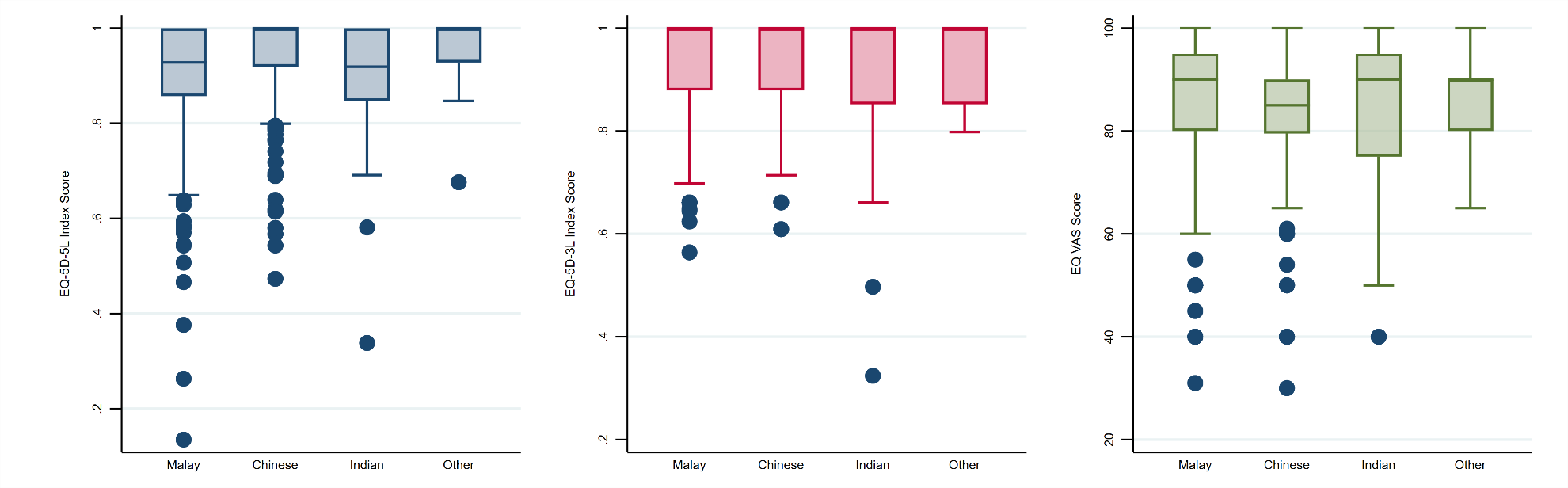


**Fig. S2 Box plots of EQ-5D-5L index score, EQ-5D-3L index score, and EQ VAS score by age group (A) and ethnicity (B)**

*Boxes show the median and interquartile range, with whiskers indicating the full range. Points represent outliers.*
